# Occult axillary lymph node metastases are of no prognostic significance in breast cancer

**DOI:** 10.1038/sj.bjc.6600070

**Published:** 2002-02-01

**Authors:** R R Millis, R Springall, A H S Lee, K Ryder, E R C Rytina, I S Fentiman

**Affiliations:** Hedley Atkins ICRF Breast Pathology Laboratory, Guy's Hospital, London SE1 9RT, UK

**Keywords:** occult metastases, micrometastases, carcinoma of breast, histological grade, tumour size

## Abstract

The significance of occult metastases in axillary lymph nodes in patients with carcinoma of the breast is controversial. Additional sections were cut from the axillary lymph nodes of 477 women with invasive carcinoma of the breast, in whom no metastases were seen on initial assessment of haematoxylin and eosin stained sections of the nodes. One section was stained with haematoxylin and eosin, and one using immunohistochemistry with two anti-epithelial antibodies (CAM5.2 and HMFG2). Occult metastases were found in 60 patients (13%). The median follow-up was 18.9 years with 153 breast cancer related deaths. There was no difference in survival between those with and those without occult metastases. Multivariate analysis, however, showed that survival was related to tumour size and histological grade. This node-negative group was compared with a second group of 202 patients who had one involved axillary node found on initial assessment of the haematoxylin and eosin sections; survival was worse in the patients in whom a nodal metastasis was found at the time of surgery. Survival was not related to the size of nodal metastases in the occult metastases and single node positive groups. Some previous studies have found a worse prognosis associated with occult metastases on univariate analysis, but the evidence that it is an independent prognostic factor on multivariate analysis is weak. We believe that the current evidence does not support the routine use of serial sections or immunohistochemistry for the detection of occult metastases in the management of lymph node negative patients, but that the traditional factors of histological grade and tumour size are useful.

*British Journal of Cancer* (2002) **86**, 396–401. DOI: 10.1038/sj/bjc/6600070 www.bjcancer.com

© 2002 The Cancer Research Campaign

## 

Axillary lymph node stage has traditionally been regarded as the most powerful prognostic indicator in invasive carcinoma of the breast. Furthermore, the greater the number of axillary node metastases, the worse the prognosis. The fact that approximately 25% of patients without involvement of the axillary nodes subsequently developed distant metastases, however, has prompted the search for other prognostic factors. One approach has been to investigate the negative lymph nodes more thoroughly in an attempt to find metastases missed by conventional assessment.

Detection of nodal metastases depends on a number of factors (Cserni, 2000). These include removal of sufficient tissue from the axilla of the patient (Reynolds *et al*, 1994), adequate dissection of the specimen (Cserni, 1998), the method of sampling the nodes for histological examination (Friedman *et al*, 1988), and the technique used to identify metastatic deposits within the histological section. In the past, it was standard practice to sample only readily palpable nodes and submit only half of each node for histology. One haematoxylin and eosin (H&E) stained section of each node was then examined microscopically. The value of axillary lymph node stage as a prognostic factor was based on studies using this methodology. Various methods for improving detection in surgical specimens have been described, such as solvents to clear axillary fat, X-ray of the specimen, or injecting radioisotope into the patient prior to surgery and then scanning the patient and the specimen. Little benefit has been demonstrated using these techniques, either in terms of yield of lymph nodes or, more importantly, detection of involved nodes (Morrow *et al*, 1984). The majority of lymph nodes can be relatively easily detected by careful palpation of the specimen. One method of increasing the sensitivity of detection of metastases is to submit more of the lymph node for histological examination, for example by cutting each lymph node larger than 5 mm into slices, and submitting all the slices for histological examination.

Other methods of detecting metastases not seen with conventional assessment include cutting additional sections, the use of immunohistochemistry employing antibodies to epithelial markers, and using polymerase chain reaction (PCR) to various RNA sequences. Such deposits have been called occult metastases and their significance is uncertain. Most of the early studies found no relationship between such metastases and prognosis. Some of the more recent studies however have found an association between occult metastases and less good prognosis. The current trend towards sentinel node biopsy has highlighted the importance of the method used to detect nodal metastases. There is now a considerable debate as to how much effort should be put into examining sentinel nodes before pronouncing them to be negative, and the significance of finding small metastases.

This study reports the prognostic significance of occult metastases detected using immunohistochemistry on axillary nodes originally considered uninvolved in a large number of patients with long follow-up, and compares them with patients with one involved node detected at initial assessment. The significance of the size of axillary nodal metastases was also investigated.

## MATERIALS AND METHODS

The patients were all treated in the Guy's Hospital Breast Unit between 1962 and 1981 for primary invasive carcinoma of the breast. Two groups were studied. The first group was composed of 477 women, who at the time of surgery had no metastases seen on histological assessment of H&E sections of the axillary lymph nodes. One slice per node was examined. All patients underwent modified radical mastectomy. Patients who were lost to follow-up within a year were excluded. Three patients received adjuvant tamoxifen, one had an ovarian ablation, and none received chemotherapy. The median follow-up was 18.9 years (range 1.0–34.8 years) in this group. There were 153 breast cancer related deaths.

The second group was composed of 202 women, who had a single involved node identified on histological assessment of H&E sections of the axillary lymph nodes at the time of surgery. All patients underwent modified radical mastectomy. Patients who were lost to follow-up within a year were excluded. Eleven patients received adjuvant tamoxifen, 11 received combined chemotherapy (cyclophosphamide, methotrexate and 5-fluorouracil) and 41 received melphalan. The median follow-up was 13.2 years (range 0.7–30.4 years) in this group. There were 111 breast cancer related deaths.

The original histological sections of the surgical specimens of the node negative patients were retrieved from the files. The primary tumours were examined for histological type, histological grade (Elston and Ellis, 1991) and the presence or absence of vascular invasion. The blocks of the lymph nodes were recut at 3 μm. One section was stained with H&E, and one using immunohistochemistry with a combination of CAM5.2 (an antibody to cytokeratins 8 and 18) and HMFG2 (an antibody to human milk fat globulin). For the immunohistochemical stains the sections were pre-digested with Trypsin. Non-specific staining was blocked using calf serum. Antibody binding was demonstrated using a streptavadin biotin technique. Positive and negative controls were included with each batch of staining.

The aim of this study was to assess the significance of occult metastases detected on further H&E sections and immunohistochemical sections. Thus we did not begin by assessing the initial H&E sections. The recut H&E sections were examined first and if they showed a metastasis, then the original H&E sections were reviewed. The immunohistochemical preparations were examined next, and where a metastasis was identified, both the recut and original H&E sections were reviewed. Occult metastases were categorised as ‘missed’ if they could be identified on the original H&E sections, as ‘definite’ if they were seen on the recut H&E sections, but not on the original H&E sections, and as ‘suspicious’ if they were seen only on the immunohistochemical sections but could not be identified on either of the H&E sections.

The size and site of all nodal deposits were noted in both the node negative (with occult metastases) and single node positive groups. Size was measured using an eyepiece graticule. Two measurements were taken; one of the greatest diameter and the other perpendicular to it, so that the involved area could be estimated by multiplying the two diameters measured. Where there was more than one deposit in a node, or more than one node involved by occult metastases, the largest deposit was measured. If multiple deposits within a node were separated by 1 mm or less, they were considered as a single deposit and measured accordingly.

The site of a metastasis was defined as parenchymal when within the node substance with or without direct spread into the sinus, capsule or through the capsule; sinusoidal when isolated deposits occurred within the subcapsular sinus or a sinus within the node; capsular or extracapsular when isolated deposits occurred within these sites. If more than one site was involved, all sites were recorded.

In the evaluation of overall survival only breast cancer related deaths were considered. Survival curves were plotted using the Kaplan–Meier method. The log rank test was used to compare univariate survival curves, and Cox's proportional hazards model was used for the multivariate analysis.

## RESULTS

### Lymph node negative patients

A total of 6851 lymph nodes were examined from 477 patients (median 14, range 1–41). Occult metastases were found in 60 patients. A single positive node was found in 55 patients, but in three patients there were two, and in two patients there were three involved nodes. The clinical and pathological features of patients with and without occult metastases are shown in Table 1

**Table 1:**
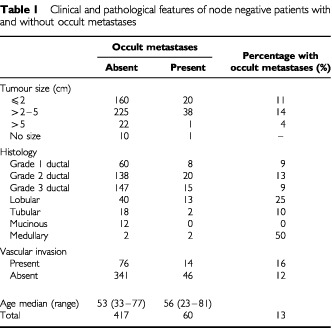
Clinical and pathological features of node negative patients with and without occult metastases

.

In 21 patients the occult metastases were detected on the recut H&E section, of which eight could then be identified in the original H&E section (‘missed’ metastases).

A further 39 patients had deposits detected on the immunohistochemical sections, of which 33 were then seen on the recut H&E sections; in two patients these were also seen on the original H&E sections (‘missed’ metastases). The metastases seen on the recut H&E sections, but not on the original H&E sections, were called ‘definite’ occult metastases.

In six patients, although positive cells were seen on the immunohistochemical preparations, they could not be identified on the H&E sections. Morphologically, however, the positive cells could be diagnosed definitely as malignant. These were termed ‘suspicious’ occult metastases. These metastases were all very small, in three cases consisting of only two or three cells, and in the other three cases consisting of five to 10 cells. In another six cases positively stained cells seen only on the immunohistochemical sections, could not be diagnosed as malignant on morphological grounds and these patients were therefore not considered to have metastases. Occult metastases were classified as missed in 10 patients, definite in 44 and suspicious in six.

The median maximum diameter of the occult metastases was 0.37 mm (range 0.01–10 mm). The missed occult metastases (median 3.8 mm, 1.8–10 mm) tended to be larger than the definite occult metastases (median 0.29 mm, 0.01–2.9 mm), *P*<0.0001, Mann–Whitney *U*), which in turn were larger than the suspicious occult metastases (median 0.01 mm, 0.01–0.03 mm), *P*=0.0003, Mann–Whitney *U*). Occult metastases were parenchymal in 25, sinusoidal in 27, both parenchymal and sinusoidal in seven, and separate deposits were seen in the peripheral sinus and the capsule in one node. All were present in nodes low in the axilla.

**Figure 1:**
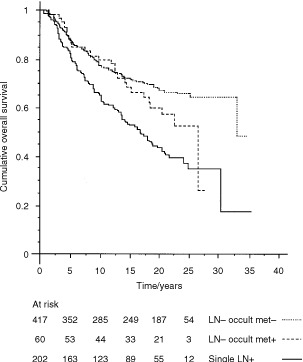
Overall survival of axillary node negative patients with and without occult metastases, and of patients with a single involved axillary node. There was no difference between node negative patients with and without occult metastases χ^2^=1.9, *P*=0.17. Survival was worse in patients with a single involved axillary node compared with the node negative group χ^2^=32.7, *P*<0.0001, and compared with the node negative patients with occult metastases χ^2^=4.1, *P*=0.04. The table underneath shows patients at risk at 5-yearly intervals.

Overall survival (Figure 1

) and relapse free survival (χ^2^=1.8, *P*=0.18) were not related to the presence of occult metastases, nor was any difference found when metastases detected in different ways (missed, definite, suspicious) were analyzed separately. Survival in the originally node negative group was however significantly related to the following prognostic factors: tumour size (*P*<0.0001), histological grade (*P*=0.005) and the presence of vascular invasion (*P*=0.01). On multivariate analysis, both tumour size (*P*=0.0003, relative risk 1.19; 95% confidence interval 1.08–1.30) and histological grade (*P*=0.03, relative risk 1.29; 95% confidence interval 1.02–1.63) were independent predictors of survival, whereas vascular invasion (*P*=0.07) and the presence of occult metastases (*P*=0.30) were not. When the presence of occult metastases was related to other pathological features, the only significant relationship was with tumour type. Patients with invasive lobular carcinoma had occult metastases more frequently than patients with invasive ductal carcinoma (25% *vs* 11%, χ^2^ with Yates correction=6.4, *P*=0.01). When patients with invasive lobular or invasive ductal carcinoma were analyzed separately, the presence of occult metastases still had no effect on survival (Lobular *P*=0.23, Ductal *P*=0.35).

### Patients with a single involved axillary node

The patients with a single positive node identified at the time of surgery had a median of 22 nodes examined (range 1–52). As seen in Figure 1 these patients had a worse survival than the node negative group. The difference in survival between patients with occult metastases and a single node positive on original assessment was also significant (χ^2^=4.1, *P*=0.04). The size of the metastases in the one-node positive patients was generally larger than that of the occult metastases (median 5.0 mm, range 0.1–30 mm, *P*<0.0001, Mann–Whitney *U*).

Some of the patients from early in the study had only a small number of lymph nodes examined, with a potential risk of understaging. Survival data were therefore reanalyzed, firstly including only patients who had at least five, and then those who had at least 10 nodes examined. The results were essentially the same as in the whole study group.

**Figure 2:**
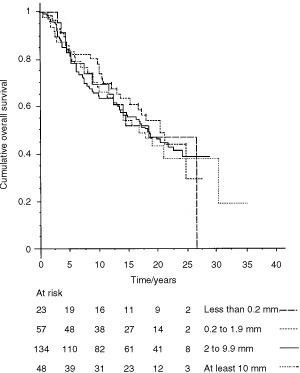
Overall survival is not related to the size of the largest axillary nodal metastasis in patients with occult metastasis or single node positive, χ^2^=0.52, *P*=0.91. Metastasis size was divided using cut-offs of 0.2, 2 and 10 mm.

Survival was not related to either the area or the largest diameter of the metastasis in the one-node positive and occult metastasis patients (Figure 2

), despite using different cut-offs. Similarly no relationship was found between the site of the metastasis and survival even after analyzing several different combinations to take into account metastases involving more than one site.

## DISCUSSION

There are two major unresolved questions regarding occult metastases. Firstly, there is no agreed definition of an occult metastasis. Secondly, the significance of such metastases is uncertain.

Several different methods can be used to identify occult metastases. Serial sections are time consuming, both for the technician cutting the sections and for the pathologist examining the slides. Immunohistochemistry for epithelial markers is much quicker to assess. Both these methods detect metastases in 10–30% of patients whose axillary specimen is negative by conventional assessment (Table 2

**Table 2:**
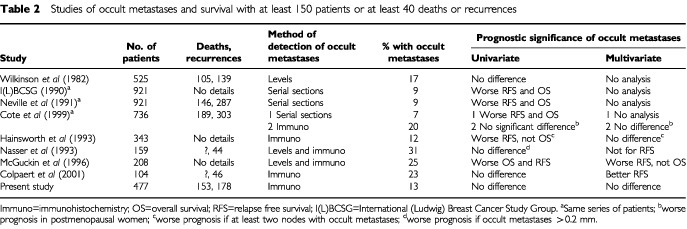
Studies of occult metastases and survival with at least 150 patients or at least 40 deaths or recurrences

). The figure of 13% for the present study is within the reported range. Reverse transcriptase polymerase chain reaction is even more sensitive, but it is difficult to find a RNA sequence that is specific to tumours; it may be necessary to use a panel of markers, particularly as the morphology of the positive cells cannot be assessed (Liefers *et al*, 1999). With such high sensitivity there is a risk of false-positive results.

There is no consensus on how nodal metastasis should be measured, with considerable variation of methods between different studies. A major problem is that two dimensional sections are used to assess a three dimensional structure. Should the area or greatest diameter be used? How should multiple metastases be assessed? Further sections of nodes with multiple apparently separate deposits might show whether they are joined or separate. Few studies address these difficulties, and many give no details of methodology. Some studies have classified nodal metastases according to size, using the term micrometastases for small deposits, but the cut-off has ranged between 0.2 and 2 mm. Although there is evidence that larger axillary nodal metastases are associated with a worse prognosis (Huvos *et al*, 1971; Fisher *et al*, 1978; Rosen *et al*, 1981; Nasser *et al*, 1993; McGuckin *et al*, 1996; Cote *et al*, 1999), this is not so in all studies (Wilkinson *et al*, 1982; Hainsworth *et al*, 1993) including the present one. Only two studies, however, included multivariate analysis. One found that the size of metastases was of prognostic significance for relapse free survival, but not for overall survival (McGuckin *et al*, 1996); the other found that metastasis size was not an independent prognostic indicator (Nasser *et al*, 1993). Subdivision of small nodal metastases into ‘isolated tumour cells’ and ‘micrometastases’ has been proposed (Hermanek *et al*, 1999). This may be useful for research, but cannot be recommended for routine use. We are unable to explain the discrepancy in the present study between the absence of a relationship between metastasis size and survival and the fact that patients with occult metastases (which tend to be smaller) have a better survival than patients with a single positive node on initial assessment.

The prognostic significance of occult metastases is controversial. Many of the early studies showed no relationship between occult metastases and prognosis, but most had inadequate numbers of deaths or recurrences for reliable evaluation of a prognostic factor (Fayers and Machin, 1995). Some of the larger studies have shown an association between occult metastases and a worse prognosis (Table 2). This effect is more marked for disease free than for overall survival, but many studies used only univariate analysis. Only one of the six larger studies found that occult metastases were an independent predictor of poor prognosis, but only for relapse free survival (Hainsworth *et al*, 1993). The largest studies, by the Ludwig group (International (Ludwig) Breast Cancer Study Group, 1990; Neville *et al*, 1991; Cote *et al*, 1999), found that, on univariate analysis, survival was worse for patients with occult metastases detected with serial sections, but not for patients with occult metastases identified with immunohistochemistry. Multivariate survival analysis was not reported for occult metastases detected with serial sections. There was worse survival associated with occult metastases identified with immunohistochemistry in post-menopausal women on multivariate analysis, but there was no such analysis of the whole study group. One other group found an effect in a different subgroup (Hainsworth *et al*, 1993).

Studies of the prognostic significance of the position of metastases within the node have produced conflicting results. Most, including the present study, found no difference (Wilkinson *et al*, 1982; Friedman *et al*, 1988; McGuckin *et al*, 1996), but a worse prognosis was associated with parenchymal metastases in one study (Nasser *et al*, 1993) and with sinusoidal metastases in another (Hartveit and Lilleng, 1996). In one study all nodes with sinusoidal metastases were found to have parenchymal metastases on deeper sections (International (Ludwig) Breast Cancer Study Group, 1990). We examined deeper sections of several nodes with sinusoidal metastases, but found parenchymal deposits only in a minority.

Immunohistochemistry detects more occult metastases than additional H&E sections, particularly those consisting of small clusters of cells or single cells. Single cells or clusters of a few cells seen only on immunohistochemistry should only be accepted as metastases if they are morphologically malignant. The nature of cytokeratin positive cells without definite features of malignancy is uncertain. Some may be malignant, but some could be the result of benign epithelial transport (Carter *et al*, 2000). Dendritic cells sometimes stain for cytokeratins, but their characteristic morphology enables these cells to be recognized. Several studies, including the present one, have shown that immunohistochemistry particularly increases the likelihood of detection of metastases associated with invasive lobular carcinoma (de Mascarel *et al*, 1992; McGuckin *et al*, 1996; Cote *et al*, 1999). Large metastases of lobular carcinoma consisting of single malignant cells scattered widely through a node can easily be missed on H&E sections. This occurred in two cases in the present study. One study suggested that immunohistochemically detected metastases were of prognostic importance in invasive ductal but not in invasive lobular carcinoma (de Mascarel *et al*, 1992), but the number of patients, particularly with lobular carcinoma, was small. A larger study found such metastases affected relapse-free survival of patients with both tumour types, although no analysis of overall survival was presented (Cote *et al*, 1999).

It is important that the relationship of survival and occult metastases is not considered in isolation. Although lymph node stage has traditionally been regarded as the most powerful prognostic indicator, recent evidence suggests that histological grade is of at least comparable prognostic importance (Elston and Ellis, 1991; Galea *et al*, 1992; Sundquist *et al*, 1999). In unselected cases tumour size is a less powerful prognostic factor, but nevertheless is of independent value on multivariate analysis. In the present study survival was associated with histological grade and primary tumour size on multivariate analysis. Four of the studies in Table 2 reported univariate survival analysis for features of the primary tumour: histological grade was significant in all four (Wilkinson *et al*, 1982; Hainsworth *et al*, 1993; McGuckin *et al*, 1996; Colpaert *et al*, 2001), tumour size in three and vascular invasion in two. Multivariate analysis was performed in four studies (Hainsworth *et al*, 1993; Nasser *et al*, 1993; McGuckin *et al*, 1996; Colpaert *et al*, 2001) with grade significant in three and size in one.

In conclusion, this study found that occult metastases in axillary lymph nodes, considered negative by conventional assessment, were of no prognostic significance. Although there are some studies which have found a difference using univariate analysis, there is little evidence that occult metastases are an independent predictor of poor prognosis. By contrast, most studies have found that histological grade, and to a lesser extent tumour size, are of prognostic value. We believe that the current evidence does not support the routine use of either multiple sections or immunohistochemistry for the detection of occult metastases in axillary clearance specimens. There is some evidence that detection of occult metastases in sentinel lymph nodes may be a useful predictor of metastases in non-sentinel nodes and thus helpful in decisions about further axillary surgery (Turner *et al*, 2000; Weaver *et al*, 2000; Kamath *et al*, 2001). However, as with prognostic factors, features of the primary tumour (such as size and vascular invasion) may also be of value. The majority of studies of occult metastases in axillary clearance specimens, including the present one, were from the period when only one slice was taken from each lymph node for histological examination. Recent guidelines from the UK breast screening programme recommend more thorough sampling of axillary lymph nodes (National Coordinating Group for Breast Screening Pathology, 1995). We suggest that any future studies of occult metastases should use large series of patients with long and complete follow-up, with modern lymph node sampling techniques, and that there should be multivariate analysis of survival including traditional histological features particularly histological grade and tumour size.
